# Insights into the structural changes that trigger receptor binding upon proteolytic activation of *Bacillus thuringiensis* Vip3Aa insecticidal protein

**DOI:** 10.1371/journal.ppat.1012765

**Published:** 2024-12-05

**Authors:** Oscar Infante, Isabel Gómez, Angel E. Pélaez-Aguilar, Luis A. Verduzco-Rosas, Rosalina García-Suárez, Blanca I. García-Gómez, Zeyu Wang, Jie Zhang, Adan Guerrero, Alejandra Bravo, Mario Soberón

**Affiliations:** 1 Departamento de Microbiología Molecular, Instituto de Biotecnología, Universidad Nacional Autónoma de México, Cuernavaca, Morelos, México; 2 State Key Laboratory for Biology of Plant Diseases and Insect Pests, Institute of Plant Protection, Chinese Academy of Agricultural Sciences, Beijing, China; 3 Laboratorio Nacional de Microscopía Avanzada, Instituto de Biotecnología, Universidad Nacional Autónoma de México, Cuernavaca, Morelos, México; University of Massachusetts Chan Medical School, UNITED STATES OF AMERICA

## Abstract

*Bacillus thuringiensis* (Bt) bacteria produce different pore forming toxins with insecticidal activity, including Cry and Vip3 proteins. While both Cry and Vip3 cause insect death by forming pores in susceptible lepidopteran larval midgut cells, their mechanisms of action differ. The Vip3Aa protoxin adopts a tetramer-structure, where each monomer has five distinct domains. Upon proteolytic activation, the Vip3 tetramer undergoes a large conformational change forming a syringe like structure that is ready for membrane insertion and pore formation. Here we show that Vip3Aa protoxin had low binding to *Spodoptera frugiperda* brush border membrane vesicles (BBMV) unlike the activated toxin that bound specifically in a concentration dependent way, suggesting that a structural change upon Vip3Aa proteolytic activation is required for efficient receptor binding. Consistently, the Vip3Aa protoxin showed no toxicity to Sf9 cells compared to the activated toxin. In contrast, Cry1Fa protoxin and its activated toxin, were both highly toxic to Sf9 cells. To identify the region of Vip3 involved in binding to BBMV proteins, different overlapping peptides from Vip3Aa covering domains III, IV and V were expressed, and binding analysis were performed against BBMV, showing that domain III is the primary binding domain. Additionally, domains III, IV and V amino acid residues that become exposed upon activation of Vip3Aa were identified. Mutagenesis of these exposed residues revealed three amino acids (K385, K526 and V529) located in two structural adjacent loops, domain III loop β5-β6 and loop α11-β16 that connects domains III and IV, that are crucial for binding to the midguts of *S*. *frugiperda* larvae and for toxicity. Our results demonstrate that proteolytic activation of Vip3Aa exposes a receptor binding region essential for its toxicity.

## Author summary

To exert toxicity, Vip3 tetrameric protoxins are activated by larval gut proteases facilitating the formation of lytic pores that destroy the midgut epithelium cells killing the susceptible larvae. Here, we demonstrate that the proteolytic activation of Vip3Aa protoxin is necessary for triggering receptor binding. Binding analysis of Vip3Aa protoxin and activated toxin to *S*. *frugiperda* midguts and toxicity assays against Sf9 cells, confirmed that proteolytic activation is necessary for an efficient binding to the insect midgut membranes and for toxicity. The analysis of residues from domains III, IV and V, that become exposed after Vip3Aa activation by proteolysis, allowed us to identify a structural binding epitope which plays an important role in receptor binding and toxicity. Our results indicate that the structural change that Vip3Aa undergoes after activation by larval midgut proteases, besides being important for membrane insertion and pore formation, also triggers receptor binding important for toxicity.

## Introduction

*Bacillus thuringiensis* (Bt) produces diverse insecticidal proteins such as Cry and Vip3 toxin with distinct structures and modes of action [1]. Among these different insecticidal proteins produced by Bt, the Cry1 toxins have been successfully used for the control of different lepidoptera insect pests for many years, either expressed in transgenic crops or included in spray formulations [2,3]. However, the evolution of insect resistance to Bt-crops in field conditions endangers the use of this technology for pest control [4]. The insecticidal Vip3 proteins are produced by certain Bt strains, shown to be highly toxic against diverse lepidopteran insect pests [5]. Vip3 are an efficient alternative to counter insect resistance to Cry1 toxins, since no-cross resistance with Cry1 toxins has been reported since Vip3 insecticidal proteins bind to different larval gut proteins than Cry1 toxins [6,7]. Nowadays, the new generation of Bt crops express simultaneously Cry1 and Vip3Aa proteins for a more efficient pest control and for countering insect resistance [8,9].

Vip3 insecticidal proteins form pores in the apical membrane of midgut cells from susceptible larvae, bursting those larval cells causing the death of the insect [10,11]. Also, it has been reported that Vip3Aa induces apoptosis in Sf9 insect cell line [12,13]. Vip3 protoxins are proteins of 90 kDa molecular weight that are activated by the larval gut proteases to yield an activated toxin composed of two fragments of 19 and a 70 kDa, respectively, that remain attached by non-covalent interactions and both fragments are required to exert toxicity [14]. The three-dimensional structure of the Vip3Aa and Vip3Bc protoxins, defined by cryo-electron microscopy (cryo-EM), revealed a tetrameric organization; each monomer is composed of five structural domains where the two N-terminal domains are involved in oligomerization and pore formation, while the remaining C-terminal domains remain exposed to the solvent [15,16]. Vip3 domain I is composed of four long α-helices which are forming part of the four-helix bundle in the oligomer, that protrudes as a stalk at the apex of the tetramer; domain II is formed by five α-helices located in the core of the oligomer and the trypsin cleavage site is located between domains I and II (between helices α-4 and α-5). Domain II show structural similarity with other pore forming domains present in other proteins, such as domain I from Cry toxins [15,16]; domain III, a β-prism formed by three antiparallel β-sheets shows structural similarity to domain II from Cry toxins, which is involved in binding to larval midgut proteins. Finally, domains IV and V, show a β-sandwich topology similar to the domain III of Cry toxins, that is also involved in binding to receptor proteins and also in structure stability [15,16]. The three-dimensional structures of Vip3Aa and Vip3Bc after protease activation, also solved by cryo-EM, revealed a large conformational change of the tetramer promoting the formation of a syringe-like structure [15,16]. The Domain I underwent a large structural rearrangement where helices α2-α3 form a long continuous helix with helix α4 and are finally assembled into a parallel four-helical coiled-coil in the lower part of the tetramer structure. It was proposed that this syringe-like structure is involved in membrane insertion and pore formation [15,16]. Although the three-dimensional structure of Vip3 protoxin and activated toxin revealed a potential pathway for triggering pore formation activity, the role of the C-terminal domains on the mode of action of Vip3 toxins, specifically in the binding interactions with membrane receptors, remains unsolved.

Here, we analyzed the binding of Vip3Aa protoxin and activated toxin to *S*. *frugiperda* brush border membrane vesicles (BBMV). We show that upon proteolytic activation of Vip3Aa, a domain III binding epitope is exposed to the solvent, allowing that the activated Vip3Aa toxin binds to BBMV, playing an important role in larval toxicity. Our results show that proteolytic activation of Vip3Aa triggers receptor binding necessary for toxicity.

## Results

### Binding of Vip3Aa protoxin and activated toxin to *S*. *frugiperda* BBMV

**Fig 1:**
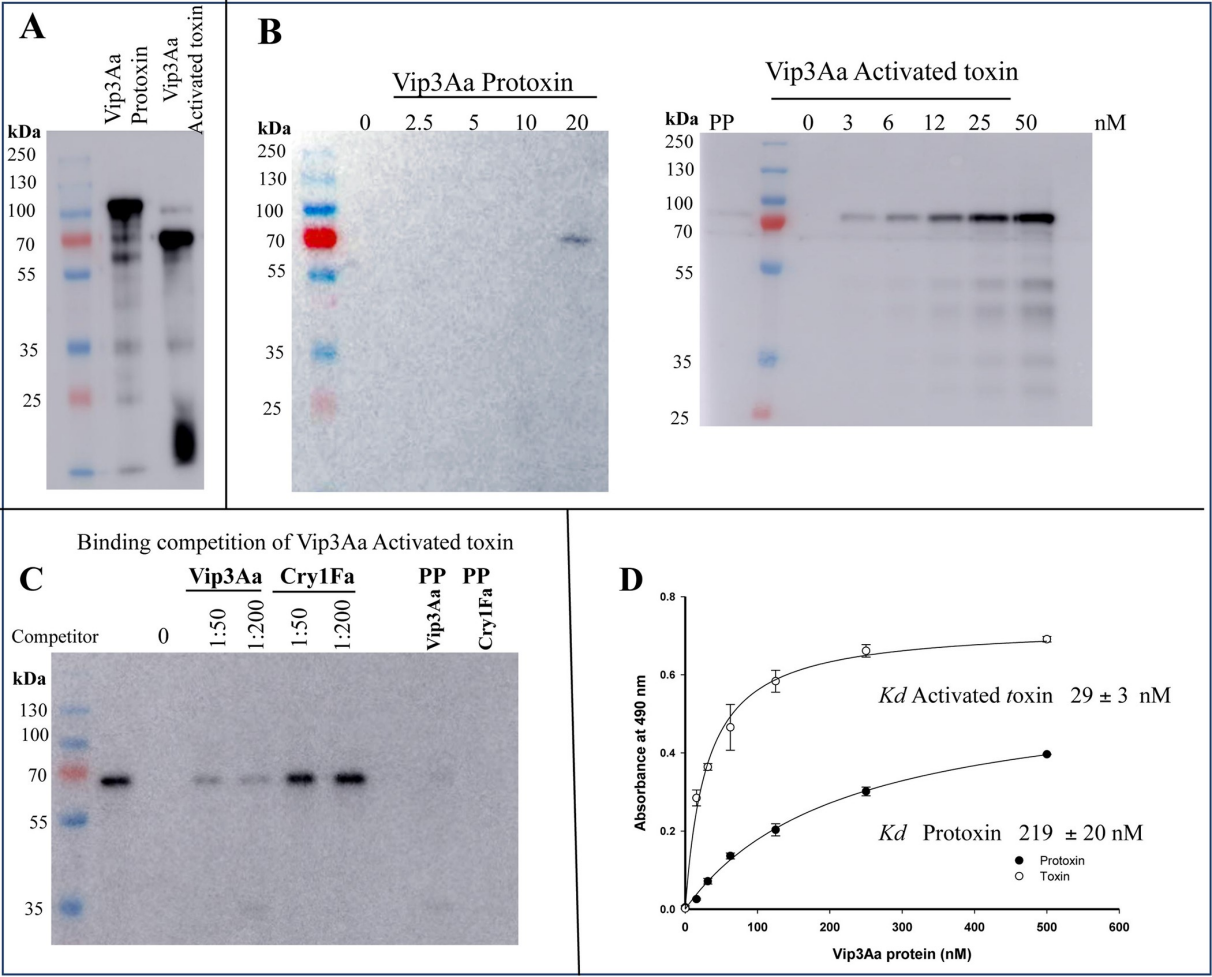
Binding of Vip3Aa protoxin or activated toxin to *S*. *frugiperda* BBMV. A, Biotin labelled Vip3Aa protoxin or activated toxins were separated in SDS-PAGE, electro-transferred to PVDF membranes and revealed with streptavidin-HRP as described in Materials and Methods. B, Binding of biotin labelled Vip3Aa proteins to BBMV isolated from 3^rd^ instar *S*. *frugiperda* larvae. Ten micrograms of BBMV protein were incubated with different molar concentrations (2.5, 5, 10 or 20 nM) of biotin labeled Vip3Aa protoxin or Vip3Aa activated protein (3, 6, 12, 25 or 50 nM) and bound toxin was recovered by centrifugation. The BBMV proteins were separated by electrophoresis in SDS-PAGE 10%, electro transfer to PVDF membranes and revealed with streptavidin-HRP to detect bound proteins as described in Materials and methods. Lanes labeled with “0” Vip3Aa are negative controls of BBMV without Vip3Aa incubation. A representative image of three replicas is shown. C, Binding competition of biotin-labeled Vip3Aa to BBMV isolated from 3^rd^ instar larvae. Ten micrograms of BBMV protein were incubated with 3 nM of biotin-labeled Vip3Aa in the absence of competitor or in the presence of 50-fold or 200-fold higher molar excess of unlabeled Vip3Aa or unlabeled Cry1Fa. Lane labeled with “0” Vip3Aa is a negative control of BBMV without Vip3Aa incubation. PP are precipitation controls of biotin labelled Vip3Aa (3 nM) with 200-fold molar excess of unlabeled Vip3Aa or Cry1Fa without BBMV. D, ELISA binding saturation assay of Vip3Aa-activated toxin or -protoxin. One μg of *S*. *frugiperda* BBMV were bound to ELISA plates and then incubated with different concentrations of Vip3Aa -protoxin or -activated toxin and bound toxins were revealed with anti-Vip3Aa rabbit antibody and with a secondary anti-rabbit antibody conjugated with horseradish peroxidase enzyme. Results are means of three repetitions.

To analyze the binding of Vip3Aa-protoxin and -activated toxin to *S*. *frugiperda* BBMV, the Vip3Aa protoxin was produced in *E*. *coli*, purified by affinity chromatography, and activated with trypsin. Both protein samples, protoxin (90 kDa protein) and activated toxin (70 kDa + 20 kDa fragments), were labelled with biotin as described in Materials and Methods (Fig 1A). Fig 1B shows that Vip3Aa-protoxin (90 kDa size) did not bind to BBMV. However, in the sample containing the highest Vip3Aa protoxin concentration, a 70 kDa band was observed. This band is not the protoxin protein, it corresponds to a contamination with activated toxin that was present in the protoxin sample (Fig 1A). In contrast, biotin-labelled activated Vip3Aa-toxin of 70 kDa bound to BBMV in a concentration dependent way (Fig 1B). To determine if the binding of Vip3Aa-activated toxin to BBMV was specific, binding competition experiments were performed with non-labelled Vip3Aa-activated toxin or with Cry1Fa-activated toxin as competitors, since it was previously shown that Cry1Fa does not share binding sites with Vip3Aa toxin [17]. Fig 1C shows that Vip3Aa-activated toxin competed the binding of biotin-labelled Vip3Aa-activated toxin to BBMV in contrast to Cry1Fa that did not compete the binding. These results confirmed that the binding of Vip3Aa-activated toxin to BBMV is specific. Finally, to confirm that Vip3Aa-protoxin does not bind to BBMV proteins, the binding of Vip3Aa-protoxin or –activated toxin to BBMV was analyzed by ELISA binding assays. In these assays, binding of protoxin or -activated toxin was revealed with an anti-Vip3Aa antibody that showed similar binding to both forms of Vip3Aa (S1 Fig). Fig 1D shows that Vip3Aa-activated toxin bound to BBMV with high affinity (*Kd* 29 +/- 3 nM) in saturable way, in contrast to Vip3Aa-protoxin (*Kd* 219 +/- 20 nM) that showed lower binding to BBMV proteins with an apparent binding affinity 7-fold lower than the Vip3Aa-activated toxin. Overall, these results show that Vip3Aa needs to be activated by proteases for an efficient binding to *S*. *frugiperda* BBMV proteins.

### Toxicity of Vip3Aa protoxin and activated toxin to Sf9 cells

**Fig 2:**
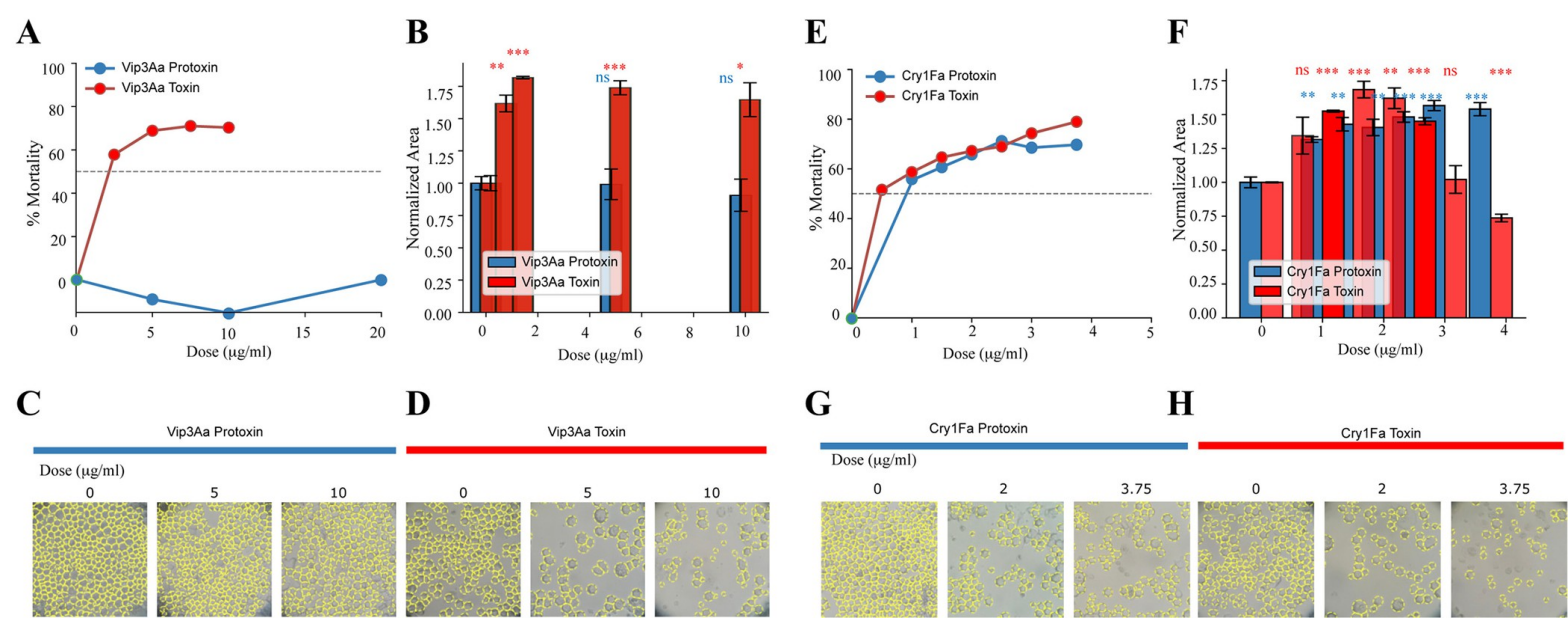
Toxicity of Vip3Aa or Cry1Fa protoxin or activated toxin to Sf9 cells. Mortality and cell swelling was determined using Cellpose3 program that allows segmentation of cell images to count cell number and cell size after treatment with different toxins. Cell images were captured 72 h post-treatment using a bright field inverted microscope. Cells were automatically identified and outlined in yellow using Cellpose3. Cell mortality was defined by the reduction of the number of cells with respect to the control and cell swelling by the increase of cell size with respect to the control as described in Materials and Methods. A, Cell mortality of Sf9 cells after exposure to different concentrations (0 and 200 μg/mL) of Vip3Aa protoxin or -activated toxin. B, Cell size of Sf9 cells treated with Vip3Aa protoxin or -activated toxin at concentrations ranging from 0 and 100 μg/mL. C, representative cell images after treatment with Vip3Aa protoxin after segmentation with Cellpose3 program. D, representative cell images after treatment with Vip3Aa activated toxin after segmentation with Cellpose3 program. E, Cell mortality of Sf9 cells after exposure to different concentrations (0 and 37.5 μg/mL) Cry1Fa protoxin or -activated toxin. F, Cell size of Sf9 cells treated with Cry1Fa protoxin or -activated toxin at concentrations in the range of 0 to 37.5 μg/mL. G, representative cell images after treatment with Cry1Fa protoxin after segmentation with Cellpose3 program. H, representative cell images after treatment with Cry1Fa activated toxin after segmentation with Cellpose3 program. Statistical significance is indicated by ***p<0.001, **p<0.01, *p<0.05, and ns for non-significant differences compared to control (t-test).

The next assay was to determine toxicity of Vip3Aa-protoxin or -activated toxin against Sf9 cells. As controls, the toxicity of Cry1Fa-protoxin or -activated toxin was also determined. It has been previously shown that large Cry1 protoxins, such as Cry1Ac or Cry1Fa, show toxicity to susceptible insect cell lines and to non-susceptible insect cell lines that were transformed with the corresponding Cry1 receptors [18,19,20]. The toxicity to Sf9 cells was analyzed by determining the number of cells and cell size using a suitable image analysis program (Cellpose3) that allows segmentation of cells allowing accurate estimation of cell numbers [21]. Cell mortality, as defined by the number of cells in relation to the controls without toxin, was determined after 72 h of treatment with different concentrations of the different proteins. The reduction in the number of cells of samples treated with toxins could be due to cell mortality or to the limitation of cell multiplication. Also, using the same image analysis tool, the cell size was determined to analyze cell swelling after toxin treatment since cell swelling has been shown to be a marker of Bt toxins cytotoxicity [19,20]. Fig 2A and 2B show that the Vip3Aa-protoxin had no effect on Sf9 cells since the total number of cells was similar to the controls and cell swelling was not observed. In contrast, the Vip3Aa-activated toxin showed significant reduction in the number of cells and cell swelling (Fig 2A and 2B). In the case of Cry1Fa, both Cry1Fa- protoxin or -activated toxin were highly toxic to Sf9 cells (Fig 2E). Also, Fig 2F shows that the cell size increased with both Cry1Fa proteins. Based on the concentration of the proteins used, these results show that Sf9 cell are more susceptible to Cry1Fa compared to Vip3Aa. Also, that both Cry1Fa protoxin or activated toxin show toxicity to Sf9 cells in contrast to Vip3Aa where the protoxin shows no toxicity compared to the activated toxin. Fig 2C, 2D, 2G and 2H shows representative images of the Sf9 cell line after treatment with the different protoxins or activated toxins as well as their segmentation with the Cellpose3 program.

### Identification of Vip3Aa binding regions to BBMV proteins

**Fig 3:**
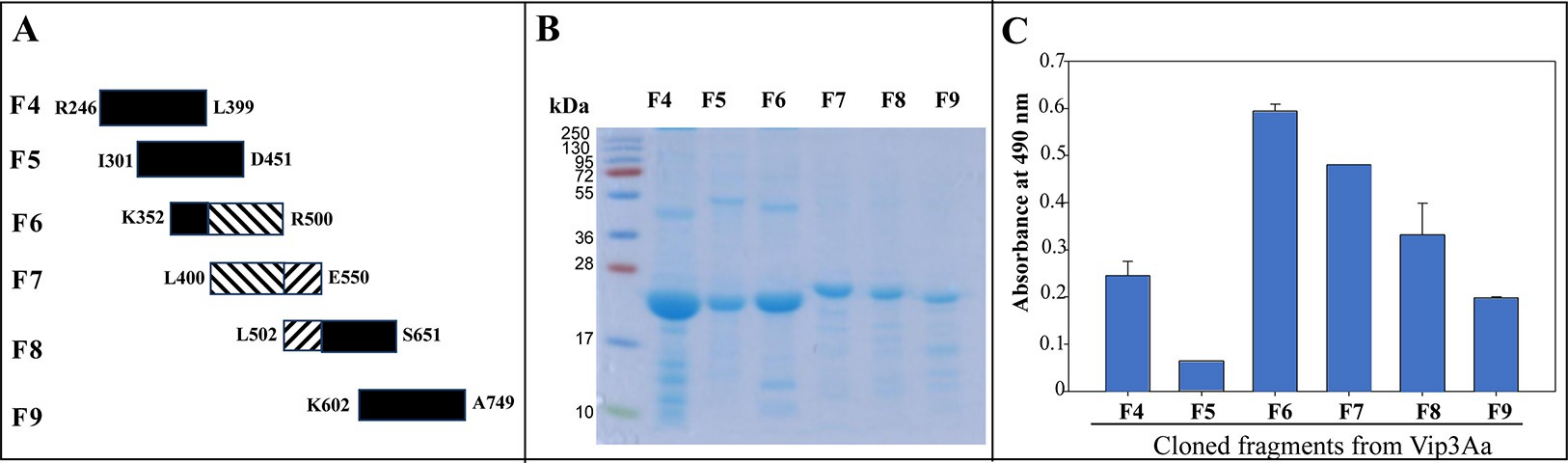
Binding of Vip3Aa overlapping fragments to *S*. *frugiperda* BBMV. A, Schematic description of the Vip3Aa overlapping fragments. B, SDS-PAGE of Vip3Aa fragments after purification as described in Materials and Methods. C, five μg of *S*. *frugiperda* BBMV were bound to ELISA plates and then incubated with 10 μg of Vip3Aa fragments F4 (R246–L399), F5 (I301–D451), F6 (K352–R500), F7 (L400–E550), F8 (L502–S651) y F9 (K602–A749). Binding of these fragments was revealed with anti-His antibody coupled to a secondary antibody coupled to HRP as described in materials and methods. The results are means of three replicas.

As mentioned previously, it was reported that Vip3Aa domains III, IV and V show some structural similarities with Cry domains II and III that are involved in receptor binding [15]. To identify regions of Vip3Aa involved in binding to its receptors in the membranes from *S*. *frugiperda*, we used a collection of previously characterized Vip3Aa fragments that were used before to map the binding regions of Vip3Aa to Cry9Aa toxin that show a synergistic interaction (F1 to F10) [22]. These fragments contain each 150 amino acids that overlap 50 to 100 amino acid residues with the next fragment. The sequence of seven of these fragments (F4 to F10) cover the Vip3Aa domain III, IV and V sequences (Fig 3A). However, in these assays, the Vip3Aa F10 fragment could not be produced and was not further analyzed. Fig 3B shows the six different Vip3Aa fragments after purification from *E*. *coli* cells. Binding of the six Vip3Aa fragments to *S*. *frugiperda* BBMV was analyzed by ELISA binding assays as described in Materials and Methods. Fig 3C shows that Vip3Aa peptide fragments F6 (K352-R500), F7 (L400-E550) and F8 (L502-S651) bound to the BBMV proteins in contrast to F4, F5 or F9 that showed lower binding to BBMV. These results suggest that at least two potential binding sites may be participating in the interaction of Vip3A with the BBMV, one binding site may be in the overlap region between F6 and F7 (L400-R500) and another binding site may be located in the overlap region between F7 and F8 (L502-E550) suggesting that domain III (N328-S532) is the main binding region in the toxin.

### Identification of Vip3Aa residues that become exposed upon proteolytic activation

Since Vip3Aa protoxin bound poorly to BBMV in comparison to the -activated toxin, we analyzed the amino acids from domains III, IV and V that become exposed upon proteolytic activation of Vip3Aa protoxin using GETAREA 1.0 beta program that calculates solvent accessible surface area of a residue in a three-dimensional structure. An amino acid residue is solvent exposed if the ratio of the surface exceeds 50% and is buried if the ratio is less than 20% [23]. According to this program, domains III, IV and V of Vip3Aa-protoxin and -activated toxin share 118 amino acid residues exposed to the solvent, where 30 residues were shown to be in domain III, 42 in domain IV and 41 in domain V. To identify residues that potentially become exposed after proteolytic activation of Vip3Aa protoxin, we looked for residues with a surface exposure ratio greater than 50% in the structure Vip3Aa-activated toxin, but less than 50% in the Vip3Aa protoxin structure, indicating a more buried location in the protoxin compared to the activated toxin. When the Vip3Aa-protoxin is activated, eight residues become exposed to the solvent. Of these eight residues that become exposed after proteolytic activation, three were located within domain III (K385, T466, N522), two residues were in the loop connecting domain III with domain IV (K526, V529), two were found in domain IV (S536, E656) and one in domain V (D751). To confirm the exposure to the solvent of these eight residues, we analyzed their exposure to the solvent using also PDBePISA program that calculates the accessible or buried surface area of residues within the three-dimensional structure of a protein showing the exposed surface of the residues in square-angstroms (Å^2^) [24]. Table 1 shows that the analysis made with both programs coincide that K385, N522, K526 and V529 become exposed to the solvent when the Vip3Aa-protoxin is activated. Also, that V529 is completely buried in the protoxin and became fully exposed in the activated toxin. In the case of T466, both programs show that this residue is partially exposed in the protoxin and became slightly more exposed in the activated toxin (Table 2). For S536, GETAREA suggested that it became exposed to the solvent, but this was not the case for PDBePISa program, that suggested that the surface exposed area of this residue was similar and partially buried in both the Vip3Aa-protoxin or -activated toxin (Table 1). Finally, in the case of E656 and N751, both programs suggest that these amino acids are partially exposed in both structures, the Vip3Aa-protoxin or -activated toxin. Thus, both programs coincide in five amino acids (K385, T466, N522, K526 and V529) from domains III and IV that become exposed to the solvent upon proteolytic activation of Vip3Aa-protoxin.

**Table 1:** Exposed residues of Vip3Aa upon protoxin activation.

Program	GETAREA- Vip3Aa Protoxin	GETAREA- Vip3Aa Toxin	PDBePISA- Vip3Aa Protoxin	PDBePISA- Vip3Aa Toxin
Residue	Surface ratio %	Surface ratio %	Å^2^	Å^2^
K385	31.2	61.2	55.6	112.97
T466	44.7	51.1	48.88	61.49
N522	43.5	76.8	58.88	107.71
K526	34.2	74.0	61.17	129.01
V529	0	75.7	2.21	96.76
S536	17.4	54.4	39.67	39.81
E656	48.7	50.0	73.13	77.43
N751	48.8	50.0	82.32	83.43

### Identification of a Vip3Aa epitope involved in binding and toxicity

**Fig 4:**
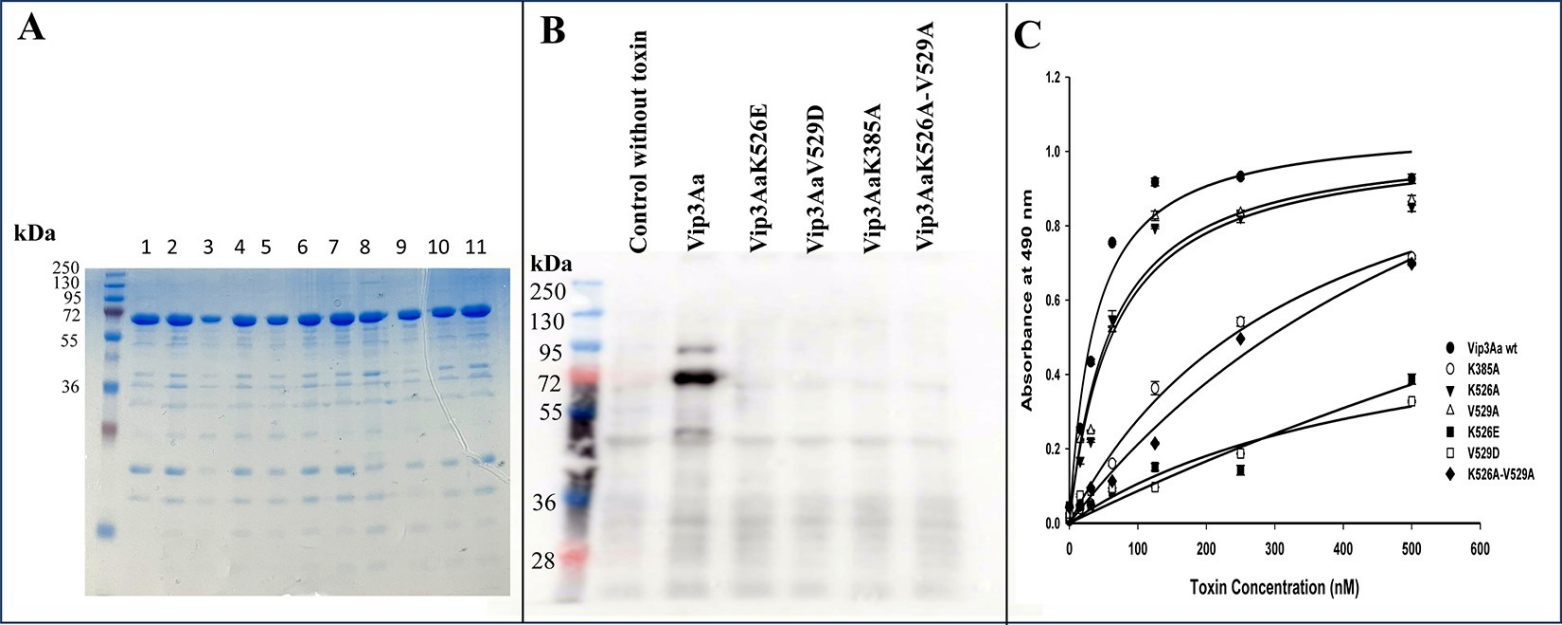
Binding of Vip3Aa and Vip3Aa mutants *to S*. *frugiperda* BBMV. A, Trypsin activation pattern of Vip3Aa (1) Vip3AaK385A (2), Vip3AaT466E (3), Vip3AaN522D (4), Vip3AaK526E (5), Vip3AaK526A (6), Vip3AaV529D (7), Vip3AaV529A (8), Vip3AaK526A-V529A (9), Vip3AaS536D (10), and Vip3AaD751R (11). A representative image of three replications is shown. B, Binding of biotinylated Vip3Aa, Vip3AaK385A, Vip3AaK526E, Vip3AaK526A, Vip3AaV529D, Vip3AaV529A and Vip3AaK526A-V529A activated toxins to *S*. *frugiperda* BBMV. The first Lane is MW marker and second lane show the negative control of BBMV incubated without Vip3Aa biotin labeled toxin. C. ELISA binding saturation assay of Vip3Aa and Vip3AaT466E, Vip3AaN522D, Vip3AaK526E, Vip3AaK526A, Vip3AaV529D, Vip3AaV529A, Vip3AaK526A-V529A, Vip3AaS536D, and Vip3AaD751R against *S*. *frugiperda* BBMV. Results are means of three repetitions.

To determine if the residues identified above are involved in binding and toxicity, single point mutations of the eight selected residues were constructed (Vip3AaK385A, Vip3AaK385E, Vip3AaT466E, Vip3AaN522D, Vip3AaK526E, Vip3AaK526A, Vip3AaV529D, Vip3AaV529A, Vip3AaS536D, Vip3AaE656K and Vip3AaD751R). In addition, since K526 and V529 are in the same loop region, a double mutant Vip3AaK526A-V529A was also constructed as described in Materials and Methods. Vip3AaE656K could not be obtained and Vip3AaK385E was not expressed in *E*. *coli*, thus these two mutations were not further analyzed. We first determine the stability of the Vip3Aa mutant proteins after trypsin activation. SDS-PAGE electrophoresis analysis showed that mutants Vip3AaK385A, Vip3AaT466E, Vip3AaN522D, Vip3AaK526E, Vip3AaK526A, Vip3AaV529D, Vip3AaV529A, Vip3AaS536D, Vip3AaE656K, Vip3AaD751R and Vip3AaK526A-V529A showed a similar activation pattern as Vip3Aa (Fig 4A). Toxicity assays of these Vip3Aa mutants against *S*. *frugiperda* larvae were performed and the median lethal concentration (LC_50_) values indicated that Vip3AaK385A, Vip3AaK526E, Vip3AaV529D showed a 73-, 78- and 66-fold reduction in their toxicity compared to Vip3Aa (Table 2). The Vip3AaK526A and Vip3AaV529A mutants showed a 10- and 6-fold reduction in toxicity, respectively. While the double Vip3AaK526A-V529A mutant showed a 20-fold reduction in toxicity compared to Vip3Aa (Table 2). Finally, mutants Vip3AaT466E, Vip3AaN522D, Vip3AaS536D and Vip3AaD751R were not affected in toxicity, showing similar LC_50_ values as Vip3Aa. These results indicate that K385, K526 and V529 residues from Vip3Aa are important for Vip3Aa toxicity.

**Table 2:** Toxicity against *S*. *frugiperda* larvae and binding affinities to BBMV of Vip3Aa and Vip3Aa mutants.

Toxin	LC_50_ in ng/cm^2^ (95% fiducial limits)	Fold-reduction of toxicity	Binding affinity (*Kd*, nM)^b^	Fold-Decrease in binding affinity
Vip3Aa	15 (11-18)		37 ± 4	
Vip3AaK385A	1100 (700-3700)	73	342 ± 47	9.2
Vip3AaT466E	13 (9-17)		ND^a^	
Vip3AaN522D	13 (8-18)		ND	
Vip3AaK526E	1180 (800-3000)	78	2410 ± 303	65
Vip3AaK526A	150 (120-180)	10	65 ± 10	1.7
Vip3AaV529D	1000 (800-2000)	66	485 ± 1	13.1
Vip3AaV529A	100 (63-140)	6	61 ± 4	1.6
Vip3AaK526A-V529A	300 (200-300)	20	715 ± 1	19.3
Vip3AaS536D	11 (7-14)		ND	
Vip3AaD751R	19 (13-26)		ND	

^a^ ND not determined

^b^ Binding affinities determined from the ELISA binding assays shown in Fig 4C

The binding to *S*. *frugiperda* BBMV of the Vip3Aa mutants that were severely affected in toxicity (Vip3AaK385A, Vip3AaK526E, Vip3AaV529D, and Vip3AaK526A-V529A) was analyzed. These mutant proteins were labeled with biotin and their binding to BBMV was analyzed by qualitative binding assays as described in Materials and Methods. Fig 4B shows that Vip3AaK526E, Vip3AaV529D, Vip3AaK526A-V529A and Vip3AaK385A mutants did not bind to BBMV in contrast to Vip3Aa that bound to the BBMV, proving that Vip3Aa K385, K526 and V529 are involved in binding to BBMV proteins. Finally, to confirm their role in the binding to BBMV, binding saturation curves of all mutants by ELISA binding assays were performed allowing the determination of apparent binding affinities (*Kd*). Fig 4C shows the binding saturation curves of the Vip3Aa mutants to BBMV, where a direct correlation on their binding affinities and the loss of toxicity of Vip3Aa mutants was found (Table 2).

**Fig 5:**
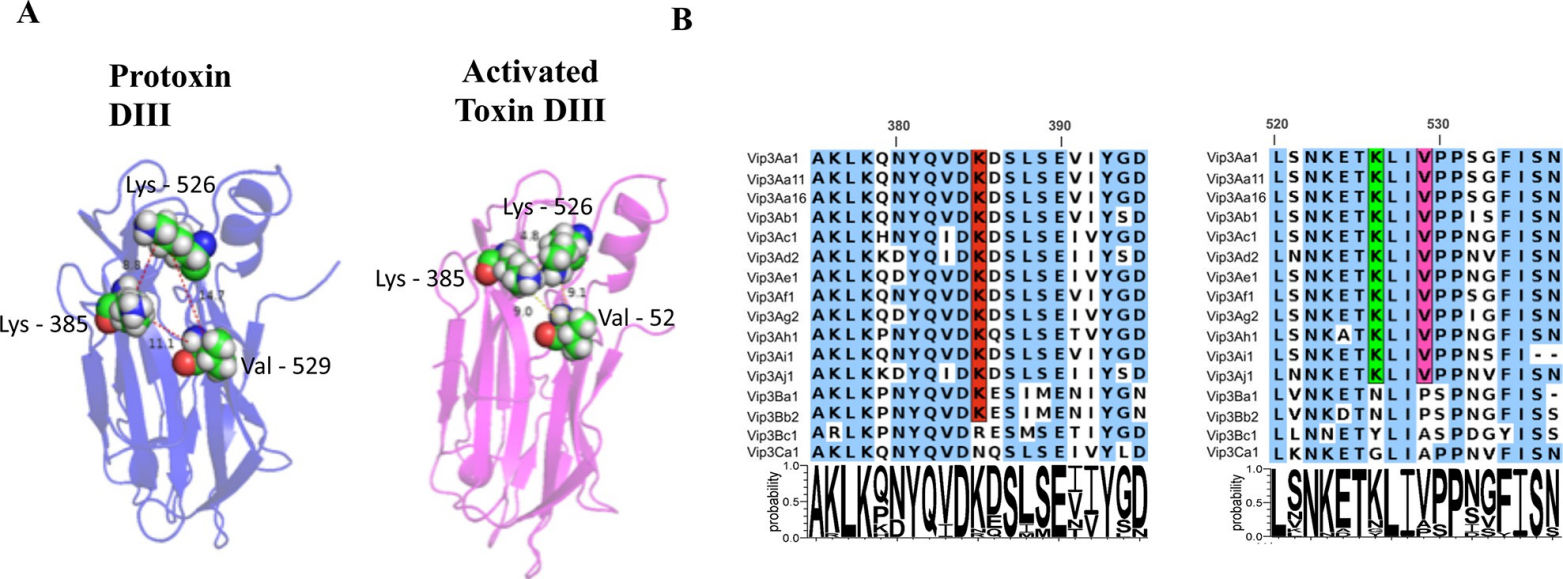
Exposed domain III residues involved in binding, after proteolytic activation of Vip3Aa protoxin. A, Vip3Aa domain III structure showing residues K385, K526 and V529 that become exposed after proteolytic activation of Vip3Aa. The epsilon-carbon for lysines, or beta-carbon for valine distances among K385 to K526 or V529 in the Vip3Aa protoxin or activated toxin are shown in the figure, showing that the distance in the protoxin structure between K385-K526 = 8.8 Å, between K385-V529 = 11.1 Å and between K526-V529 =14.7 Å, while in the activated toxin are K385-K526 = 4.8 Å, K385-V529= 9.0 Å and K526-V529=9.1 Å. B, Vip3 Domain III amino acid sequence alignment. The Vip3Aa K385, K526 and V529 residues are highlighted inside the rectangles.

Vip3Aa K385 is contained in F6 fragment, while K526 and V529 are contained in F7 and F8 fragments described above. When we localized these residues in the 3D-structure of Vip3Aa, we found that these three residues are in two structurally adjacent loop regions. Specifically, residue K385 is in domain III loop β5-β6, while the other two residues K526 and V529 are found in loop α11-β16, connecting domain III with domain IV (Fig 5A). The molecular distance between K385 to K526 or to V529 in the Vip3Aa-protoxin or the -activated toxin, showed that residue K385 approaches K526 upon proteolytic activation of Vip3Aa, since the distance of the epsilon-carbons of these Lys residues, showed a reduction in distance from 8.8 to 4.3Å (Fig 5A), supporting a potential structural binding region.

To determine if this Vip3Aa binding epitope identified here is conserved among the different Vip3 proteins, a multiple sequence alignment was performed using the amino acid sequences of domain III from different Vip3 proteins. Fig 5B shows that the Vip3Aa K385, K526 and V529 are highly conserved within all Vip3A members. In the case of Vip3B proteins, the Vip3Bc shows a R residue instead of K385, while residues K526 and V529 are not conserved in Vip3B proteins. Finally, in Vip3Ca these three residues are not conserved (Fig 5B).

## Discussion

It has been suggested that proteolytic activation of Vip3Aa insecticidal protein is a necessary step to exert insecticidal activity [25]. In agreement, it was shown that Vip3Bc-protoxin shows no pore-formation activity in contrast to Vip3Bc-activated toxin that showed high pore formation activity in synthetic liposomes vesicles [16]. We show here that Vip3Aa-protoxin shows very low or no toxicity to Sf9 cells in contrast to Vip3Aa-activated toxin that showed higher toxicity (Fig 2). To assess cell mortality, we used the cell image Cellpose3 program that allowed segmentation of cells allowing to determine accurately the number of cells and the cell size of each cell after toxin treatment by analyzing dozens of cell images making an accurate estimation of the toxicity of the different Vip3Aa and Cry1Fa proteins analyzed. However, other reports have shown variable toxicity levels of Vip3A-protoxins to insect cell lines [12,13,26,27,28,29]. The main problem in these studies resides principally in the high variability in Vip3Aa- toxicity assays, resulting sometimes in lack of sustained results. For instance, the toxicity of Vip3Aa protoxin to Sf9 cells, that have been reported by the same group in different reports, showed mortality values that ranged from 25 to 60% depending on the dose of the protoxin, exposure time and procedure to determine toxicity [12,13,28,29]. Interestingly, in some reports when these authors compared toxicity of Vip3A protoxin to the Vip3Aa-activated toxin, they showed that Vip3A activated protein was more effective to kill Sf9 cells than the protoxin, showing mortality values for the activated toxin that ranged from 55 to 82% [25,29]. Similar results were also observed for Vip3Af proteins when analyzed against Sf21 cells, where the Vip3Af-protoxin showed lower mortality (up to 35%) compared to the Vip3Af-activated toxin showing 82% mortality of the cells [26]. The different mortality rates of the Vip3-protoxin observed in these reports as well as the low mortality of Vip3Aa-protoxin shown here (Fig 2), could be due to the different assays used to assess cell mortality or to the fact that the different protoxin samples assayed could be contaminated with activated toxins, thus the presence of different amounts of activated toxin could explain some of these results, as was previously discussed [25]. Nevertheless, our results are consistent with the published data, showing a similar trend where the Vip3A-activated toxin shows higher toxicity than the Vip3Aa-protoxin to insect cell lines. These results are consistent with the hypothesis that Vip3-protoxins needs to be activated to exert their toxicity.

We hypothesized here that the lower mortality observed with Vip3Aa-protoxin could be related to lower binding capacity of Vip3Aa-protoxin compared to the -activated toxin. To probe this hypothesis, we compared the binding of these two forms of Vip3Aa to *S*. *fruigiperda* BBMV showing that Vip3Aa-protoxin shows low binding to BBMV compared to Vip3Aa-activated toxin that bound specifically to BBMV (Fig 1). These results support that proteolytic activation of Vip3Aa triggers binding to insect gut Vip3Aa receptors. Vip3 and Cry toxins have distinct modes of action, since they bind to different larval gut proteins, resulting in no cross-resistance among these insecticidal proteins [7,17]. In agreement, we show that Vip3Aa-activated toxin binding to BBMV was competed by Vip3Aa but not by Cry1Fa, confirming that Vip3Aa and Cry1Fa do not share binding sites as previously reported (Fig 1C) [17]. In addition, we show here that Cry1Fa and Vip3Aa show a distinct mode of action, since Sf9 cells were highly susceptible to Cry1Fa-protoxin in contrast to Vip3Aa-protoxin that showed no toxicity to Sf9 cells (Fig 2). It was previously shown that Cry1Ab-protoxin binds its cadherin receptor through domain II exposed loops, indicating that proteolytic activation of Cry1Ab is not a necessary step required for receptor binding [30,31]. Also, the C-terminal region of Cry1Ab-protoxin was able to bind to alkaline-phosphatase and aminopeptidase-N receptors, suggesting that this region contains additional binding sites involved in Cry-protoxin mechanism of action [31]. In contrast, we show here that proteolytic activation of Vip3Aa is necessary for its efficient binding to BBMV. These results suggest that, although Vip3 and Cry toxins show some structural similarities, they have different modes of action leading to pore formation and toxicity.

The structural similarity of Vip3Aa domains III, IV and V with Cry domain II and III, that are involved in binding to larval midgut proteins, suggested that these Vip3Aa domains could be involved in binding to BBMV proteins [15,16]. The analysis of binding to *S*. *frugiperda* BBMV of different Vip3Aa peptide fragments comprising the different Vip3Aa domains revealed that domains II-III of Vip3Aa were involved in binding to BBMV proteins, while domain V was involved in binding to the peritrophic membrane [29]. In agreement, a similar analysis performed with Vip3Af protein, showed that domain III was involved in binding to *S*. *frugiperda* BBMV, but domains IV and V were not [26]. However, analysis of a Vip3A chimera, where the last 190 amino acids, corresponding to half domain IV and complete domain V from Vip3Ac were replaced by those of Vip3Aa (Vip3AcAa), showed that the resulting Vip3AcAa chimera was toxic to *Ostrinia nubilalis* larvae while the parental toxins were not toxic against these larvae, suggesting that domain IV and V are somehow involved in insect specificity of Vip3 toxins [32]. Here, we have analyzed the binding of different Vip3Aa overlapping peptide fragments to *S*. *frugiperda* BBMV allowing us to narrow a Vip3Aa binding region located Vip3Aa domain III (Fig 3) confirming that Vip3A domain III is the major binding domain as was previously reported [26,29].

Since Vip3Aa-protoxin shows low binding to larval BBMV in contrast to the Vip3Aa-activated toxin, we speculated that an important binding region of Vip3Aa become exposed after activation by midgut proteases. The analysis of the published structures of the Vip3Aa- protoxin and -activated toxin [15] revealed eight amino acid residues of domains III, IV and V that become exposed to the solvent upon protoxin activation (Table 1). However, additional comparison analysis of the exposure of these eight residues, showed that only five become exposed to the solvent upon proteolytic activation of Vip3Aa protoxin (Table 1). These exposed resides in the Vip3Aa-activated toxin mapped in domain III and in the loop connecting domains III-IV. By site directed mutagenesis of these amino acid residues we demonstrated that K385 located in domain III loop β5-β6 and K526 and V529 located in loop α11-β16 connecting domain III and domain IV are involved in binding to BBMV proteins and toxicity against *S*. *frugiperda* larvae. The reduced toxicity of these Vip3Aa mutants was not due to instability to protease treatment since all mutants showed a similar activation pattern as Vip3Aa. Interestingly, K385 residue seems to play a major role in this binding epitope, since alanine substitutions of K385 gave the largest reduction in toxicity (73-fold) compared to K526 or V529 (10-fold or 6-fold reduction of toxicity) respectively (Table 2). Only when K526 or V529 were substituted by charged residues (K526E or V529D), or when both amino acids were mutated simultaneously a greater loss of toxicity compared to Vip3Aa was observed (78-fold, 66-fold or 20-fold respectively) (Table 2). In agreement, the reduction of the binding affinities of mutations in these residues showed a direct correlation between the loss of binding and the reduction in toxicity (Table 2). However, the double mutant Vip3AaK526A-V529A showed a substantial decrease in binding affinity (19-fold) which correlated with 20-fold decrease in toxicity while Vip3AaK385A mutant the binding affinity to BBMV proteins was decreased 9-fold while the toxicity was decreased 73-fold indicating also that K385 plays a major role in this binding interaction. By comparing the structures Vip3Aa-protoxin and Vip3Aa-activated toxin it can be observed that both K385 and K526 side chains are closer in the activated toxin structure (Fig 5A), suggesting that activation of Vip3Aa results in a proper structure of this binding epitope allowing receptor binding. It is likely that other residues besides the three residues that become exposed to the solvent are involved in binding. Further mutagenic studies of neighbor residues or other regions of the toxin are required to fully map all binding epitopes involved in Vip3Aa toxin action. Recently it was shown that Vip3Aa protoxin and activated toxin share binding sites in *S*. *exigua* BBMV [33]. This suggest that the binding region identified here contributes for an efficient high affinity binding to the Vip3Aa receptor.

Recently, it was shown that other exposed Vip3Aa domain III loops, β4-β5 and β14-β15, are involved in binding to BBMV and toxicity to *S*. *frugiperda* larvae [34]. These loops were identified due to the structural similarity of Vip3 domain III with Cry toxins domain II, where Cry domain II exposed loops, located in the vertex of the β-prism, are involved in binding to receptors and toxicity [3]. The β4-β5 loop is contained in Vip3Aa fragment F6 while the β14-β15 is contained in fragment F7 explaining in part their higher binding to BBMV proteins (Fig 3). Interestingly, the binding epitope of Vip3Aa identified here is in the upper part of the domain III β-prism (Fig 5A). Since two binding domain III regions are involved in receptor binding, we hypothesized that a two-binding step mechanism could be involved in Vip3Aa binding to its midgut receptor (s). The binding epitope identified here is exposed to the solvent upon protoxin activation allowing receptor binding in contrast to β4-β5 and β14-β15 that are exposed to the solvent in both Vip3Aa- protoxin or -activated toxin. Thus, we propose that the binding epitope identified here is involved in a first binding event with the Vip3Aa receptor, allowing then the binding of other toxin regions, such as the loops β4 -β5 and β14 -β15 from domain III to the receptor(s). This remains to be analyzed.

Various members within the Vip3 protein family show differential toxicity levels against different lepidopteran pests [5,10]. The three amino acids described here, that become exposed in the activated toxin, are conserved in all Vip3A proteins, partially in Vip3B but not in Vip3C proteins. In the case of Vip3B proteins, residue K385 is conserved in Vip3Ba1, Vip3Bb2 while Vip3Bc1 has an Arg residue in this position, indicating a consistent positive charge in this position in the three Vip3B proteins, while residues corresponding to K526A or V529 are not conserved in Vip3B proteins (Fig 5B). Since the three dimensional structures of Vip3Bc protoxin and activated toxin have been reported [16], we analyzed the exposed residues of Vip3Bc loops β5-β6 (393QVDRESMSET402) and α11-β16 (539TDLLNNETYLIASPDGY555) upon proteolytic activation (Table 3). Our analysis revealed that Vip3Bc loop β5-β6 has not a major structural change upon proteolytic activation. However, Vip3Bc R396, which corresponds to Vip3Aa K385, is exposed in both protoxin or activated toxin suggesting that it may be involved in receptor binding (Table 3). In the case of Vip3Bc loop α11-β16 three amino acids (Y547, I549 and A550) become exposed in the activated Vip3Bc toxin. Interestingly, in the amino acid alignment (Fig 5B) Y547 in Vip3Bc corresponds to Vip3Aa K526 and A550 in Vip3Bc corresponds to Vip3Aa V529 suggesting a similar structural change in Vip3Bc. Future mutagenesis analysis of these residues in Vip3Bc will determine if this region is involved in binding and toxicity as is the case of Vip3Aa. These data could explain in part the differential toxicity of the different Vip3 proteins against distinct lepidopteran species, although further studies remain to be done.

**Table 3:** Exposed residues of Vip3Bc loops β5-β6 and α11-β16 upon protoxin activation.

Program	GETAREA- Vip3Bc Protoxin	GETAREA- Vip3Bc Toxin	PDBePISA- Vip3ABc Protoxin	PDBePISA- Vip3Bc Toxin
Residue	Surface ratio %	Surface ratio %	Å^2^	Å^2^
R396	62.5	88.5	135.32	167.49
L537	39.3	52.0	57.53	77.14
N544	45.1	56.4	58.36	87.16
Y547	36.8	52.9	72.83	106.16
I549	7.5	56.2	15.87	57.87
A550	20.5	100	21.78	69.41

These results show that, upon activation by midgut proteases, a binding epitope becomes exposed to the solvent, allowing receptor binding and toxicity. Since the activated toxin change dramatically its conformation, which is proposed to be ready for membrane insertion, this conformation becomes also capable for receptor binding. The identification of Vip3Aa receptor is still a controversial since some insect proteins such as the Scavenger receptor-C, fibroblast growth factor receptor-like protein and Prohibitin II have been proposed to serve as Vip3Aa receptors by using Sf9 cells as model system to fish Vip3Aa binding proteins [27,28]. In addition, down regulation of a transcription factor and a knock-out mutation in a chitin synthase gene had been associated with *S*. *frugiperda* resistance to Vip3Aa [35,36]. However, in the case of Scavenger receptor-C and fibroblast growth factor receptor-like protein, knock out mutants of *S*. *frugiperda* constructed by CRISPR/CAS9 revealed that these are not functional receptors of Vip3Aa in the larvae [37]. Thus, it still is pending the identification of the insect larval receptor(s) that are involved in Vip3Aa membrane insertion and toxicity. Also, the definition of the role of domains IV and V in Vip3Aa mode of action remains unsolved. The identification of Vip3 binding regions that are crucial for receptor interaction could facilitate the engineering of Vip3Aa toxins for enhanced toxicity against certain insect pests and also to develop strategies for countering resistance to these insecticidal proteins.

## Materials and methods

### Strains and materials

*Escherichia coli* BL21/pET28b-*vip3Aa11*strain, containing full-length gene of *vip3Aa11* (GenBank accession number AAR36859) was previously described [22]. In the case of Cry1Fa, a pHT315-Cry1Fa plasmid transformed into Bt 407 acrystalliferous strain [38] was used for Cry1Fa production. *S*. *frugiperda* ovarian Sf9 cells were maintained and propagated in Sf-900 II SFM medium (Gibco) at 27°C. A previously characterized *S*. *frugiperda* population Sf-UAEM1 was maintained at the Instituto de Biotecnología UNAM without exposure to Bt toxins at 25°C with 75% relative humidity and a light-dark photoperiod of 13:11 h [39].

### Analysis of Vip3Aa and Vip3Bc exposed residues

The exposed residues of Vip3Aa protoxin (PDB ID: 6TFJ), Vip3Aa activated toxin (PDB ID: 6TFK), Vip3Bc protoxin (PDB ID: 6YRF) or Vip3Bc activated toxin (PDB ID: 6RFG) were analyzed by using two different programs, the Program GETAREA 1.0 beta for calculating solvent accessible surface area of protein structures (https://curie.utmb.edu/GET.html) and program PDBePISA (https://www.ebi.ac.uk/pdbe/pisa/). GETAREA calculates "The "random coil" value of a residue X which is the average solvent-accessible surface area of X in the tripeptide Gly-X-Gly in an ensemble of 30 random conformations [23]. In the case of PDBePISA, the program that calculates the accessible/buried surface area as square-angstroms (Å^2^) of a residue within a protein three-dimensional structure [24]. The exposed residues between both structures were compared to identify residues that become exposed after proteolytic activation of Vip3Aa.

### Multiple sequence alignment

The sequences used were the following: Vip3Aa1 (NCBI accession number: AAC37036.1), Vip3Aa11 (NCBI accession number: AAR36859.1), Vip3Aa16 (NCBI accession number: AAW65132.1), Vip3Ab1 (NCBI accession number: AAW65132.1), NCBI accession: AAR40284), Vip3Ac1 (NCBI accession number: ABL23218.1), Vip3Ad2 (NCBI accession number: CAI43276.1), Vip3Ae1 (NCBI accession number: CAI43277.1), Vip3Af1 (NCBI accession number: CAI43277.1), NCBI accession: CAI43275.1), Vip3Ag2 (NCBI accession number: ACL97352.2), Vip3Ah1 (NCBI accession number: ABH10614.1), Vip3Ai1 (NCBI accession number: AGU13858.1), Vip3Aj1 (NCBI accession number: AIT93172.1), Vip3Ba1 (NCBI accession number: AAV70653.1), Vip3Bb2 (NCBI accession number: ABO30520.1), Vip3Bc1 (PDB accession number: 6YRF) and Vip3Ca1 (NCBI accession number: ADZ46178.1). For the proteins belonging to Vip3A, domain 3 was selected from amino acids 325 to 536 [15]. For the Vip3B proteins, a scrutiny was carried out on the three-dimensional structure of the Vip3Bc1 protein, and it was determined that domain 3 constitutes amino acids 341 to 557 [16]. Finally, for the Vip3Ca1 protein, since it does not have a structure, domain III was selected in the same way as the Vip3B proteins. Vip3 Domain 3 sequences were aligned using the ClustalW 2.1 program, (https://www.genome.jp/tools-bin/clustalw), with the Pairwise Alignment parameter set to SLOW/ACCURATE, and the rest of the parameters default. The alignment was visualized using Jalview version 2.11.4.0 (https://www.jalview.org/). Finally, a logo of the sequence was made using the WebLogo 3 program version 3.7.12 (https://weblogo.threeplusone.com/create.cgi).

### Preparation of brush border membrane vesicles (BBMV)

Midgut tissues from *S*. *frugiperda* third instar larvae were dissected and stored at -70°C. BBMV were prepared by differential precipitation using MgCl_2_ as previously described [40]. Enrichment of BBMV was calculated by measuring the aminopeptidase-N specific activity in comparison to midgut homogenates as previously described [38].

### Vip3Aa site directed mutagenesis

Plasmid pET28a-*vip3Aa11* [22] was used as template to perform site directed mutagenesis by using the QuickChange mutagenesis kit from Thermofisher Scientific (Waltham, MA), following the manufacturer’s instructions. Table 4 shows the nucleotide sequence of the mutagenic oligonucleotides that were synthesized at the Instituto de Biotecnología UNAM facilities. The mutated plasmids were transformed to *E*. *coli* DH5α blue strain and transformant strains were selected in LB-kanamycin 50 μg/mL. Plasmids were purified by a DNA extraction Wizard®PLUS SV kit (Promega, Madison WI) and sequenced at the Instituto de Biotecnología, UNAM facilities. The Vip3Aa mutated plasmids were then transformed into BL21 strain for protein production.

**Table 4:** Oligonucleotides used for site-directed mutagenesis.

Oligonucleotide	Sequence
K385E	5´-TTATCAAGTCGATGAGGATTCCTTATCGG-3´
K385A	5´-TTATCAAGTCGATGCGGATTCCTTATCGGAAG-3´
T466E	5´-AGCGGAGTATAGAGAGTTAAGTGCTAATGATG-3´
N522D	5´-GCTAGCAACAGACTTAAGCGATAAAGAAAC-3´
K526E	5´-AGCAATAAAGAAACTGAATTGATCGTCCCGCC-3´
Vip K526A	5´-TTAAGCAATAAAGAAACTGCG TTGATCGTCCCGCCAAG-3´´
V529D	5´-AAGAAACTAAATTGATCGACCCGCCAAGTG-3´
Vip V529A	5´-AAAGAAACTAAATTGATCGCGCCGCCAAGTGG-3´
S536D	5´-GCCAAGTGGTTTTATTGACAATATTGTAGAGAACGGG-3´
E656K	5´-AAGTCAAAATGGAGATAAAGCTTGGGGAG-3´
D751R	5´-AGCGGTGCTAAACGTGTTTCTGAAATG-3´
K526AV529A	5´-ATAAAGAAACTGCATTGATCGCCCCGCCAAGTG-3´

### Purification of Vip3Aa proteins and Vip3Aa overlapping fragments

For production of Vip3Aa or Vip3Aa mutants, a 50 mL culture of BL21/pET28a-*vip3Aa11*strain or BL21 containing mutant plasmids (pET28a-*vip3AaK385E;* pET28a-*vip3Aa*K385A; pET28a-*vip3AaT466E*; *pET28a-vip3AaN522D*; *pET28a-vip3AaK526E*; *pET28a-vip3AaK526A*; *pET28a-vip3AaV529D*; *pET28a-vip3AaV529A*; *pET28a-vip3AaK526A-V529A; pET28a-vip3Aa S536D*; *pET28a-vip3Aa E656K*) were grown in Luria-Bertani (LB) medium supplemented with 50 μg/mL kanamycin until an absorbance of 0.6 was observed at 600 nm and induced by the addition of 1 mM isopropylthio-β-galactoside (IPTG) for 5 h at 30°C with agitation at 200 rpm. Then, cells were collected by centrifugation at 10,000 *xg* for 8 min and the cell pellet was suspended in 10 mL of phosphate-buffered saline pH 7.4 (PBS). Cells were lysed by sonication for 5 min (70% power, 3 sec pulse on, 5 sec pulse off) and centrifuged at 13,000 *xg* for 15 min at 4°C.

The cloning of ten Vip3Aa overlapping fragments covering Vip3Aa sequence in plasmid pET28b was previously described [22]. Vip3Aa fragments covering Vip3Aa domains III, IV and V: F4 (R246–L399), F5 (I301–D451), F6 (K352–R500), F7 (L400–E550), F8 (L502–S651) and F9 (K602–A749) were used in this study. To produce Vip3Aa fragments, a 30 mL culture of BL21/pET28b-F4 to F9 strains were grown in 2xTY medium supplemented with 50 μg/mL kanamycin until an absorbance of 0.6 was observed at 600 nm and induced by the addition of 1 mM IPTG overnight at 30°C with agitation at 200 rpm. The cells were collected by centrifugation and sonicated as described above for Vip3Aa.

Proteins were purified by Ni-affinity chromatography (GE Healthcare Biosciences, Uppsala, Sweden), pre-equilibrated with PBS buffer. Proteins bound to the column were eluted using the elution buffer (20 mM Tris-HCl, 500 mM NaCl, 250 mM imidazole, pH 8.0). Vip3Aa protoxin was dialyzed against PBS and proteolytically activated with 1:50 trypsin (Sigma) (Trypsin/Protoxin) at 37°C for 2 h.

### Purification of Cry1Fa

Bt 407/pHT315-Cry1Fa strain was grown in nutrient sporulation medium plates [41] supplemented with 10 μg/mL erythromycin for 72 h at 30°C until sporulation was completed. The spore/crystals suspensions were solubilized in 5 mM of NaOH with 800 rpm agitation at 37°C for 2 h and proteolytically activated with 1:30 trypsin (Sigma) (Trypsin/Protoxin) at 37°C for 2 h. Protein concentration of protoxins and toxins was determined by the Bradford method using BSA as a standard.

### Cytotoxicity assay of Cry1Fa and Vip3Aa protoxins and toxins to Sf9 insect cell line

The Sf9 cells were seeded into 96-well culture plates with a density of 2x10^4^ cells/well in a volume of 100 μL for four h. Both toxins and protoxins were diluted (Vip3Aa in 1X PBS pH 7.4 and Cry1Fa in 5 mM NaOH) to obtain 6.25, 12.5, 25, 50, 75, 100 μg/mL and 50, 100, 200 μg/mL doses for Vip3Aa activated toxin and protoxin, respectively, and 10, 15, 20, 25, 30, 37.5 μg/mL for Cry1Fa protoxin or activated toxin. The dilution solution of each toxin was used as negative control. Different doses in a volume of 25 μL was added to Sf9 cells. The bioassays were monitored for 72 h using a bright field inverted microscope (60X/1.4NA) with a cellphone (iPhone 7) mounted on the ocular for digital image capture. Images were processed for segmentation using the Cellpose3 [21], specifically the super-generalist "cyto3" model, version 3 (February 2024), adept at identifying cell boundaries in low-quality images (doi: https://doi.org/10.1101/2024.02.10.579780, https://github.com/MouseLand/cellpose/). The parameters employed included `model_type="cyto3"`, `diameter=0`, `channels=[0,0]`, and `niter=2000`.

Post-processing, cell images were analyzed, and regions of interest (cells) were quantified for cell number and size for each treatment group. Mortality rates were calculated using a Python script that compared treated cell groups against controls. The control group, with no toxin exposure, served as the baseline. Mortality rate for each concentration was calculated as:

$$Mortality\;Rate\;(\%)=\left(1-\frac{Average\;Treated\;Cells}{Average\;Control\;Cells}\right)\times 1000$$

Cell size was determined by measuring the area of each cell identified by the Cellpose3 model, with average cell size calculated as:

$$Average\;Cell\;Size=\frac{Areas\;of\;Cells}{Number\;of\;Cells}$$

Measurement errors were quantified through standard error. Scripts were written in Python using libraries such as Pandas for data manipulation, and Matplotlib and Seaborn for visualizing dose-response relationships. Statistical analyses were conducted using the t-test to compare cell sizes between control and treated groups, categorizing results based on their p-values: *** for p-values below 0.001, ** for below 0.01, * for below 0.05, and ns for 0.05 or above.

### ELISA binding assays

ELISA plates (Nunc) were coated with BBMV (5 μg in 100 μL PBS) overnight at 4°C. ELISA plates were washed with PBS-T 0.05 (PBS pH 7.4 with Tween-20 0.05%). Ten μg of Vip3Aa fragments in 100 μl PBS-T were incubated with the coated ELISA plates for 1 h at 37°C. Plates were washed three times with PBS-T 0.05 and bound fragments were detected using rabbit anti-His HRP (1: 7500) antibody (QIAGEN). Finally, 0.5 mg/mL *o*-phenylenediamine (Sigma) and 0.05% H_2_O_2_ were used as substrates for peroxidase activity detection. Reaction was stopped by adding 50 μL of 1 M H_2_SO_4_ and absorbance was measured at 490 nm using an ELISA microplate reader. For the detection of Vip3Aa protoxin or activated toxin, ELISA plates (Nunc) were coated with one μg of protoxin or activated toxin as described above and detected with anti-Vip3Aa antibody (1: 20000) that was previously described [22] and a secondary antibody anti-rabbit HRP (1: 25000) (Thermo). Finally, peroxidase activity detection and revealed as described above.

For ELISA binding saturation assays, ELISA plates were coated with BBMV (1 μg in 100 μL PBS) as described above and incubated with different molar concentrations of Vip3Aa-activated, -protoxin or Vip3Aa activated mutants in 100 μl PBS-T and incubated with the coated ELISA plates for 1 h at 37°C. Bound proteins were detected using rabbit anti-Vip3Aa (1:10,000) antibody and with the secondary anti-rabbit antibody (1:10,000) conjugated with horseradish peroxidase enzyme and revealed as described above. Results are means of three repetitions. Comparison of binding data were analyzed by t-test using GraphPad Prism 75.0b.

### Qualitative binding assays

Vip3Aa protoxin or activated toxin were labelled with Biotin. The biotinylation was carried out with the Biotin Labeling Kit (Roche) following the manufacturer’s instructions. Briefly, 40 μL of biotin was used for each mg of protein and incubated for 1 h at room temperature. Excess unbound biotin was removed by size exclusion chromatography using sephadex G-25. The biotinylation of the proteins was confirmed by western blot. Fifty ng of protein were separated by 10% SDS-PAGE and electro-transferred to PVDF membrane. The membrane was washed twice with PBS and subsequently incubated for 20 min with PBS-T 2 (PBS pH 7.4 with 2% Tween 20), followed by two washes with PBS-T 0.1 (PBS pH 7.4 with 0.1% Tween 20). The membrane was incubated with Streptavidin-Horseradish Peroxidase (HRP) Conjugate for 1 h and washed twice in PBS-T 0.1. Detection was performed with the Western Blotting Luminol Reagent kit (Santa Cruz Biotechnology) in an Amersham Imager 600 device (GE Healthcare Life Sciences, Little Chalfont, UK).

For qualitative binding assays, 10 μg of BBMV were mixed with different molar concentrations of Vip3A protoxin or activated toxin in 100 μL of binding buffer (PBS pH 7.4 0.1% Bovine Serum Albumin, 0.05% Tween 20) and incubated for 45 min at room temperature. Membrane pellet was recovered by centrifugation at 13,000 *xg* for 15 min at 4°C. The BBMV pellet was washed twice with binding buffer and the BBMV pellet were separated by 10% SDS-PAGE and electro-transferred to PVDF membrane. The biotin labeled proteins were revealed with Streptavidin-HRP as described above.

### Toxicity assays against *S*. *frugiperda* larvae

For bioassays, Vip3Aa protoxin was induced as described above and the concentration of the 90 kDa protoxin in the homogenate was estimated by separation in 10% SDS-PAGE along with different known concentrations of BSA. The concentration of the Vip3Aa protoxin (90 kDa band) was estimated using imageJ software (https://imagej.net/ij/index.html). *S*. *frugiperda* larvae were grown on diet previously described [42]. Different doses of Vip3Aa protoxin, from 0.1 ng to 500 ng, were applied to the diet surface in 24-well polystyrene plates (Cell Wells; Corning Glass Works, Corning, NY). One first instar larva per well, and one 24-well plate was used per dose of Vip3Aa protoxin or Vip3Aa protoxin mutants (making a total of 144 larvae per sample). The plates were incubated at 28°C with 65% ± 5% relative humidity and a 16-h light/8-h dark cycle. Dead larvae were recorded after 7 days, larvae were considered dead if they remained the size of neonates or if no movement was observed after molesting the larvae. The 50% lethal concentration (LC_50_) values were calculated by Probit software using Polo Plus Probit and Logit Analysis version 1.0 LeOra software.

## Supporting information

S1 Fig Anti-Vip3Aa antibody recognizes both the Vip3Aa protoxin and activated toxin. ELISA binding assay of Vip3Aa protoxin and Vip3Aa activated toxin using the anti-Vip3Aa antibody as described in Materials and Methods. Results are means of three repetitions.
(TIF)
