# Correction: Insights into the structural changes that trigger receptor binding upon proteolytic activation of *Bacillus thuringiensis* Vip3Aa insecticidal protein

**DOI:** 10.1371/journal.ppat.1014366

**Published:** 2026-06-23

**Authors:** Oscar Infante, Isabel Gómez, Angel E. Pélaez-Aguilar, Luis A. Verduzco-Rosas, Rosalina García-Suárez, Blanca I. García-Gómez, Zeyu Wang, Jie Zhang, Adan Guerrero, Alejandra Bravo, Mario Soberón

In the Funding statement, the grant number from the funder DGAPA/PAPIIT is listed incorrectly. The correct grant number is: IN202623.
