# Supplementary figures and images for: Insights into the structural changes that trigger receptor binding upon proteolytic activation of Bacillus thuringiensis Vip3Aa insecticidal protein
Source: PLoS Pathog. 2024 Dec 5;20(12):e1012765. doi: 10.1371/journal.ppat.1012765 (PMC11651543; doi:10.1371/journal.ppat.1012765)

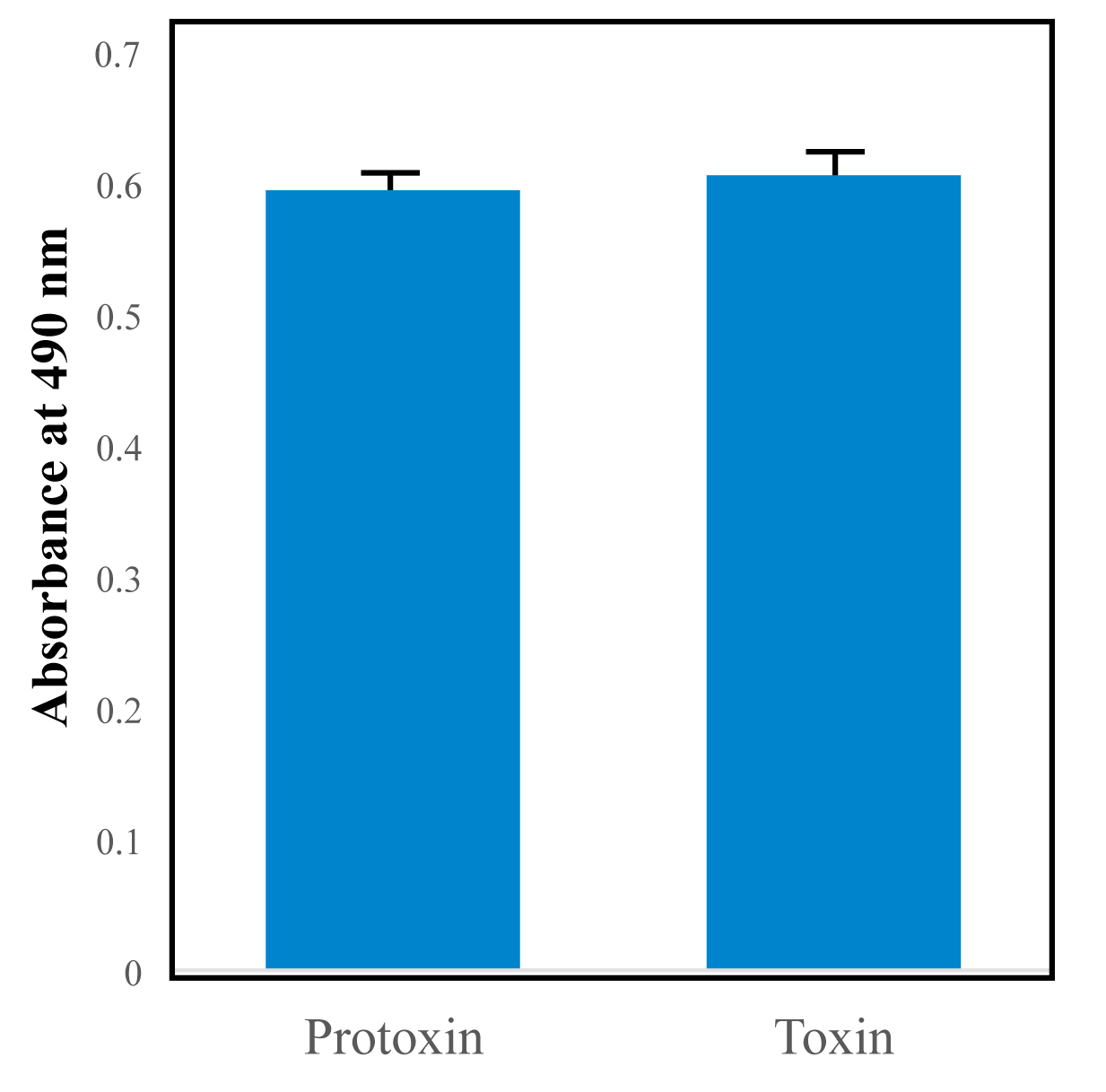

Supplement: S1 Fig — ELISA binding assay of Vip3Aa protoxin and Vip3Aa activated toxin using the anti-Vip3Aa antibody as described in Materials and Methods. Results are means of three repetitions. (TIF) [file ppat.1012765.s001.tif]
